# 100% Fruit Juice and Dental Health: A Systematic Review of the Literature

**DOI:** 10.3389/fpubh.2019.00190

**Published:** 2019-07-12

**Authors:** DeAnn Liska, Michael Kelley, Eunice Mah

**Affiliations:** ^1^Biofortis, Mérieux NutriSciences, Addison, IL, United States; ^2^Michael Kelley Nutrition Consulting, Chicago, IL, United States

**Keywords:** caries, erosion, sugar, teeth, oral, sugar-sweetened beverage

## Abstract

**Background:** The objective of this systematic review was to assess the scope and breadth of publicly available prospective cohort and randomized controlled trial (RCT) literature on 100% fruit juice and dental caries or tooth erosion in humans.

**Methods:** We performed a systematic search in MEDLINE/PubMed, EMBASE, and Web of Science for studies published from inception through May 2018, and the Cochrane Library databases for reports published through January 2018. Prospective cohort studies or RCTs conducted on dental health and 100% fruit juice, and published in English were selected. No restrictions were set for age, sex, geographic location, or socioeconomic status.

**Results:** Eight publications representing five independent prospective cohort studies and nine publications on nine RCTs were included. All prospective cohort studies were in children or adolescents, and all RCTs were in adults. Prospective cohort studies on tooth erosion found no association between juice intake and tooth erosion, while those on dental caries incidence reported either no association or an inverse association between 100% fruit juice intakes and dental caries incidence. RCTs on tooth erosion showed decreased microhardness, increased surface enamel loss, increased erosion depth, greater enamel softening, and/or increased pellicle layer with 100% fruit juice, and those on dental caries showed increased demineralization of enamel slabs with 100% fruit juice.

**Conclusions:** The existing evidence on 100% fruit juice intake and caries and tooth erosion are not conclusive. Overall, prospective cohort studies in children and adolescents found no association between 100% fruit juice intake and tooth erosion or dental caries, but, RCT data in adults suggests that 100% fruit juice could contribute to tooth erosion and dental caries. The RCT data, however, were from small, short-term studies that utilized intra-oral devices generally devoid of normal plaque or saliva action, and generally employed conditions that are not reflective of normal juice consumption.

## Introduction

In the United States, dental caries and tooth erosion affect 19 and 30% of school-going children, respectively (1, 2) while 32% of adults have untreated dental caries (1). Dental caries and tooth erosion have been indicated as the outcomes most related to food intake and dietary practices and have, therefore, been assessed by many authoritative organizations as part of dietary recommendations, primarily with respect to sugar (3, 4). For example, the 2015 Dietary Guidelines for Americans included dental caries as one of the chronic health conditions assessed with respect to added sugars (5). The committee relied on a review of sugars and dental caries conducted for the World Health Organization (WHO) (6, 7), which combined data on total, free, added, and non-milk extrinsic sugars (8). Likewise, the Scientific Advisory Committee on Nutrition (SACN) in the United Kingdom has also included oral health as a primary outcome in its scientific report on carbohydrates and health (9), and included all sources of sugars and polysaccharides, such as polyols, sugar-added beverages, and fruit and fruit juices in its assessment.

Dental caries refers to damage to the hard surfaces of teeth. The dental caries process is complex and many factors play a role in the risk and prevalence of caries in a population. In general, the etiology of dental caries involves three main factors: (1) presence of cariogenic microorganisms, (2) exposure to fermentable substrates (e.g., carbohydrates), and (3) a susceptible tooth surface/host (10). Cariogenic bacteria produce organic acids from fermentable substrates, mainly sugars and starches, and these acids can damage enamel through demineralization to cause the loss of enamel. Because sugars and starches are commonly found within the foods and beverages of many societies, the risk of developing dental caries extends to nearly all individuals. In the absence of fermentable substrates, the pH within dental plaque is close to neutral. Saliva contains calcium, phosphorus, and bicarbonate and the calcium and phosphorus can contribute to the hydroxyapatite structure of enamel, while the bicarbonate functions to return and maintain plaque pH above 5.7. Remineralization of enamel can occur following transient demineralization, and thus, remineralization and demineralization are in a dynamic balance with the progression of caries occurring when the effects of demineralization predominate. A number of factors can modify caries risk, including eating behavior, socioeconomic status, birth weight, genetics, and age (10).

Tooth wear is also a concern, as it increases the risk of caries and other dental health issues. Tooth wear increases with age and is also influenced by modifiable factors, most notably diet (9, 11, 12). Three types of tooth wear are recognized: abrasion, attrition, and erosion. Abrasion and attrition occur from physical forces, whereas erosion occurs from demineralization. Tooth erosion is defined as the progressive, irreversible loss of dental hard tissues by a chemical process without bacterial involvement. Erosion is differentiated from demineralization associated with caries as it is caused by exogenous acid, most commonly from foods or beverages. The process of demineralization is the same as described for caries, but the acid supplied by foods or beverages affects a much larger surface area of enamel, while the acid effects in the caries process are localized where the plaque exists. Thus, dental erosion is often found in areas that are plaque-free, whereas dental caries are in sites of plaque accumulation.

As mentioned earlier, dental health, particularly caries, have been included as an outcome related to intakes of sugar-containing foods and beverages. Much of the concern has focused on the content of either naturally occurring free sugars or added sugars. However, foods fitting this category are heterogeneous. In particular, 100% fruit and vegetable juices, as well as whole fruits and vegetables, at times have been included in this category. For example, the majority of reports used for policy assessment have combined data from sugar-sweetened beverages, fruit drinks, whole fruits, desserts, confectioneries, and 100% fruit juice (8, 9, 13). Specifically, data for 100% fruit juice, which provides nutrients expressed from the fruit, has often been combined with data on sugar-sweetened beverages and/or foods devoid of these nutrients.

The validity of combining 100% fruit juice with other sugar-sweetened beverages has been recently questioned in light of reports suggesting that 100% fruit juice may function differently with respect to dental health. For example, Salas et al. (14) conducted a meta-analysis on tooth erosion and diet and reported that the association of caries with natural fruit juices (OR, 1.20; 95% CI 0.02–1.42) was lower than that of carbonated beverages (OR, 1.60; 95% CI, 1.29-1.99) and sports drinks (OR, 2.13; 95% CI, 0.95–4.77), based on cross-sectional and prospective cohort data (14). Further, the 2015 Dietary Guidelines for Americans (DGA) specifically recommended the consumption of 100% fruit juice, and not fruit drinks (15). Additionally, the American Academy of Pediatrics (AAP) concluded that 100% fresh or reconstituted fruit juice can be part of a healthy diet of children older than 1 year, whereas fruit drinks are not considered nutritionally equivalent and are not recommended (16). However, it is not clear whether available evidence supports limiting or eliminating the intake of 100% fruit juice based on possible effects on dental health. Thus, the objective of this systematic review was to assess the scope and breadth of the publicly available prospective cohort and randomized controlled trial (RCT) research literature on 100% fruit juice and dental caries or tooth erosion in humans.

## Methods

### Standards

The search strategy and selection process was conducted according to the Preferred Reporting Items for Systematic Reviews and Meta-Analysis (PRISMA) guidelines (17). The study was registered on PROSPERO (an international prospective register of systematic reviews) prior to data extraction (ID: CRD42018095619).

### Search Strategy

Comprehensive literature searches were conducted independently in three databases (MEDLINE/PubMed, EMBASE, Web of Science [WOS]) for reports published from inception through the date of the search (initial search conducted on January 3, 2018 with an updated search conducted on May 7, 2018). The Cochrane Library database (http://www.cochranelibrary.com/about/central-landing-page.html) was also searched from inception through January 20, 2018. Search terms included “juice or juices” and terms related to dental health (including oral, tooth, teeth, dental, caries, and cavities) and beverage(s). The search included the use of terms for fruits as well as sugar-sweetened beverages to assure coverage of studies with beverages and dental health (full search terms for each database are provided in Supplementary Table S1). Searches were specific to literature published in English and in humans, but were not restricted to any age or publication date ranges. Hand-searching of selected reviews and publications was also conducted. Specifically, the studies presented in the SACN report were included in the hand-searching, with searches on cohorts conducted in-depth to identify data on 100% fruit juice and dental health.

### Literature Selection

Selected studies were restricted to peer-reviewed human intervention and prospective cohort studies that were relevant to the general population with no age, sex, socioeconomic status or geographic limitations. Studies conducted in populations with a defined systemic disease were excluded, but assessment of a periodontal disease as an outcome was not an exclusion criterion. However, if the study was conducted specifically in individuals with established gum disease, the study was excluded. Reviews, editorials, *in vitro* studies, animal studies, and non-peer-reviewed reports were not included. Only publications in English were selected. In addition, consistent with the criteria in the SACN report, single meal studies as well as studies in preterm infants, or those with combinatorial interventions (e.g., drugs, toothpaste, behavioral) were also excluded (9). Observational studies other than prospective cohorts (e.g., cross-sectional, case-control, case series) were also excluded consistent with the SACN report and recommendations from the Food and Drug Administration and National Evidence Library (18, 19) due to the low level of evidence provided by these types of studies.

Although the focus was 100% fruit juice consumed as a beverage, the initial selection (abstract searching) was not restricted to indication of 100% fruit juice given the potential that study publications reporting on sugar-sweetened beverage, particularly in cohort analyses, may include data on 100% fruit juice in the body of the article or supplemental materials. If these studies included a relevant endpoint and met the study design criteria, the full-text and other publically-available information were further screened for data specifically on 100% fruit juice.

### Outcome Measures

Outcomes for dental health were consistent with the SACN report (9) and included dental caries and tooth erosion. In addition, outcomes of tooth mineralization/ demineralization were also included, as these processes are part of the mechanism of dental caries and tooth erosion, and are measured in short-term clinical studies. The oral health outcomes of periodontal disease and oral cancer were not included.

Dental caries outcomes included mineralization/ demineralization of caries lesions and/or white spot lesions, as well as standard dental health indices. The standard approaches to assessment of dental caries include the Decayed, Missing, or Filled Teeth (DMFT or dmft when assessing permanent dentition or deciduous dentition, respectively) Index used for decay to crown of the tooth only, as well as the Decayed, Missing, or Filled tooth Surfaces (DMFS or dmfs when assessing permanent dentition or deciduous dentition, respectively) Index of decay activity that is valid when a large number of teeth are present (9, 10, 20). The International Caries Assessment and Detection System (ICDAS), which uses a 7-point scoring range, is designed to give gradation of severity of carious lesions, and radiological scoring and is used to detect the amount of demineralization of the enamel and/or dentin from a dental radiograph (9, 10, 20). Direct assessment of dental caries by a dentist is also used as a general measure. Tooth erosion is most often measured by microscopy and/or visual exam, and reported with respect to the amount of loss of the surface enamel, underlying dentin, and pulp exposure and several indices exist, with the overall measure the amount of tooth surface loss (9, 11).

### Data Selection, Extraction, and Analysis

Title and abstract screenings were conducted by two independent investigators (DL, EM), and if discrepancies occurred in the title/abstract screen, the records were included in the full-text search listing for resolution. The full-texts of potentially eligible studies were retrieved and assessed for confirmation of meeting inclusion, and not meeting any exclusion criteria independently by two investigators (DL, EM). Disagreements between the two investigators were resolved by discussion. The rationale for selection and exclusion were documented in detail.

Extraction of data from selected studies included population, intervention, control, and outcome (PICO) chart and were performed by two investigators (DL, MK). Populations and outcome definitions and methods, as well as background factors were documented in detail for comparison of study designs. In addition, consideration of statistical approaches and noted confounder included in analyses were also documented.

### Risk of Bias Assessment

Risk of bias for RCTs was performed using Version 2.0 of the Cochrane risk-of-bias tool for randomized trials (i.e., RoB 2) (https://sites.google.com/site/riskofbiastool/welcome/rob-2-0-tool?authuser=0). One non-randomized study [i.e., (21)] was assessed using the ROBINS-I, tool which was developed to assess risk of bias in the results of non-randomized studies that compare health effects of two or more interventions included in Cochrane Reviews (https://sites.google.com/site/riskofbiastool/welcome/home?authuser=0). Finally, prospective cohort studies were assessed using the Newcastle-Ottawa Scale (NOS), which is an assessment tool that is also recommended by the Cochrane Collaboration (22). All quality assessments were independently performed by two investigators (DL, EM), whereby disagreements were discussed and resolved prior to finalization of the ratings.

## Results

### Search and Selection

**Figure 1:**
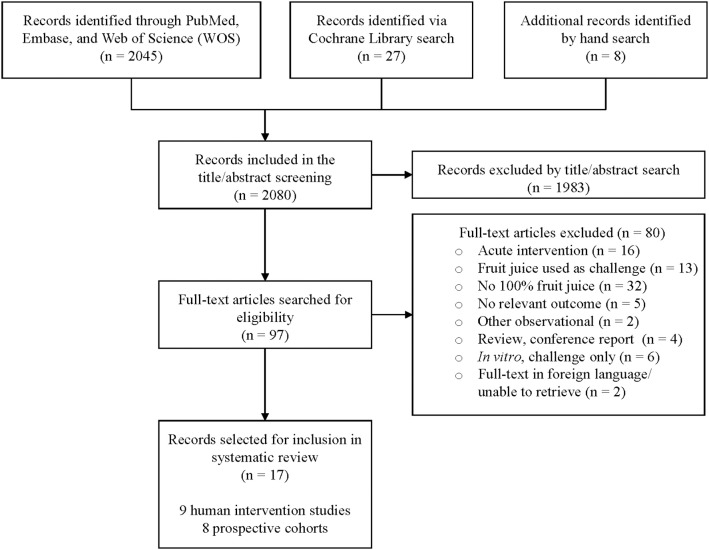
PRISMA diagram.

The findings of the literature search-and-select are summarized in the PRISMA diagram (Figure 1). Search findings from databases and hand-searching were combined and duplicate records removed prior to title and abstract screening, resulting in 2,080 records. After title and abstract screening, 1983 records were excluded (no beverage or relevant oral health outcome, *n* = 1,675; non-relevant reviews, *n* = 66; animal or *in vitro* study, *n* = 147; not a prospective observational study or RCT, *n* = 95). Full-text publications were obtained for the remaining records (*n* = 97). The 97 full-text publications were screened in detail against the prospectively defined inclusion/exclusion criteria, and 17 studies were included in this analysis, with 80 excluded (Figure 1). A listing of the 80 excluded studies with rationale for exclusion per study is provided in Supplementary Tables S2a,b.

### Prospective Cohort Studies

Eight publications identified for inclusion were prospective cohort studies. These publications represented seven different studies on five independent cohorts in children or adolescents that included fruit juice and a relevant dental health outcome. Descriptions of these studies, including NOS quality ratings, are provided in Table 1, and the results of the studies are summarized in Table 2. Two of these studies (20, 29) did not clearly identify 100% fruit juice, but were included in the Salas et al. review (14) as representing data on natural fruit juices separate from fruit drinks and other sugar-containing beverages. The overall dataset includes three reports on the Iowa Fluoride Study cohort (23, 26, 27, 29), and one report each from the Low Income African American cohort in Michigan (28), the Low Income African American Cohort in Alabama (20), a Leicestershire UK cohort (24), and a cohort consisting of children in a small community in the Netherlands (25). Two of these cohorts (the Iowa Fluoride cohort and the Low Income African American cohort in Michigan) were also included in the SACN report, another two (the Leicestershire UK cohort and the Netherland children cohort) were reported in the Salas et al. (14) review, while the fifth cohort (the Low Income African American Cohort in Alabama) appears not to have been included in previous systematic reviews. Three studies reported on erosion and juice (23–25) and four reported on caries and juice (20, 26–29). Using the NOS tool, four studies (23, 25, 27, 28) were rated as Good, one study (20) was rated as Fair, and the remaining three studies (24, 26, 29) were rated as Poor. Studies rated as Poor had low quality for the outcome/exposure domain mainly due to unclear description of blinding of outcome assessment and inadequate follow-up of cohorts.

**Table 1:** Description of prospective cohort studies on 100% fruit juice and dental health.

**References**	**Cohort**	**Age at baseline**	**Follow-up duration**	**Dental assessment measure**	**Oral health factors addressed**	**Analysis approach**	**Dietary assessment**	**100% Fruit juice details**	**Funding**	**NOS rating**
**EROSION**										
Warren et al. (23)	Iowa Fluoride Study, USA	Birth	5 y	Evaluation of tooth impressions by one examiner using published criteria, with independent examination of subsample by second examiner	None reported	*t*-test, bivariate Chi-square and Pearson analyses. No confounders included.	Diet diaries every 1–6 mo assessed for consumption events/d	100% FJ identified separately from other beverages and fruit drinks	Not reported	Good
Dugmore and Rock (24)^*^	Leicestershire Schools, UK	12 y	2 y	Trained and calibrated examiner scored prevalence of erosion, including area and depth of lesions	Tooth cleaning, gingival health, plaque index, calculus	Multifactorial analysis adjusted for all variables, paired and unpaired *t*-test, where appropriate, Chi-squared test for strength of associations	Questionnaire, details not provided	Reported FJ, with separate category for “fizzy pop,” details not provided	Not reported	Poor
El Aidi et al. (25)^*^	Netherlands	10–12 y	3 y	Two trained and calibrated examiners scored incidence and progression of erosion	Tooth brushing, tooth grinding, swishing, straw use, plaque, salivary flow, pH	Multivariate analysis, unadjusted but included assessment of interaction of food/drink, tooth grinding, and dietary habits	FFQ	Reported as FJ with a separate category for carbonated soft drinks, details on inclusion of fruit drinks vs. 100% juice not provided	Industry & University Grant funding	Good
**CARIES AND MINERALIZATION**										
Marshall et al. (26, 27)	Iowa Fluoride Study, USA	Birth	5 y	Caries assessed by duplicate examinations from trained examiners; inter-rater variability assessed and reported	None reported	Multivariate, included time/age, dentition type, fluoride intake	Diet diaries every 1-6 mo assessed for consumption events/d	100% FJ identified separately from other beverages and fruit drinks	Government Grant Funding	Good Poor
Lim et al. (28)	Detroit Dental Health Project, USA	Up to 5 y	2 y	Single exam per subject by one of four dentists trained and assessed for reliability with the International Caries Detection and Assessment System (ICDAS)	Tooth brushing, dental visits, caregiver oral health	Adjusted for sample weights and controlled for child's age, total sugar intake, baseline caries, and caregiver's oral health behaviors; Imputed values for missing numbers	Trained interviewers used the 2000 Block Kids FFQ	3 beverage categories: 100% FJ, milk, and soft drinks, which included fruit drinks	Government Grant Funding	Good
Chankanka et al. (29)	Iowa Fluoride Study, USA	1.5 mo	5, 9, and 13 y	Caries assessed by duplicate examinations from trained examiners; inter-rater variability assessed and reported	Fluoride exposure, tooth brushing frequency	Multivariate, included time/age/ dentition type, SES, and oral health factors	Diet diaries every 1–6 mo assessed for consumption events/d	100% FJ identified separately from other beverages and fruit drinks	Not reported	Poor
Ghazal et al. (20)	Alabama High-Caries Risk African-American Children, USA	1 y	3 y	Oral exams conducted by one of three calibrated dentists with assessment of inter- and intra-examiner reliability	Tooth brushing, provided oral hygiene kit, fluoride	Multivariate analysis with adjustment for age	Caregivers answered detailed questionnaire	100% juice assessed separately from other beverages	Government Grant Funding	Fair

*d, day; FFQ, Food Frequency Questionnaire; FJ, fruit juice; mo, months; NOS, Newcastle-Ottawa Scale; SES, socio-economic status; y, years*.

* Despite unclear description of fruit juice, these studies have been included in a previous meta-analysis (14) and were categorized as providing “natural fruit juice.”

**Table 2:** Results of prospective cohorts on 100% fruit juice and dental health.

**References**	**Analysis cohort (age, N)**	**Dentition type**	**100% FJ exposure**	**Erosion results**	**Caries results**	**Conclusions**
**EROSION**						
Warren et al. (23)	5 y, *N =* 355	Late deciduous	Not reported	No statistically significant relationships between tooth wear and juice consumption at any time point or with any cumulative measure (detail not provided)	n/a	No association of juice with tooth wear.
Dugmore and Rock (24)	12 y, *N =* 1,753; 14 y, *N =* 1,149	Permanent	Not reported	FJ was not a significant factor in the logistic regression for affecting prevalence of tooth erosion at 14 y (data not provided) Only cross-sectional data in 12 y-olds included in the publication; OR = 1.42 (95% CI, 1.08–1.85), *P =* 0.011	n/a	No association was found in the prospective assessment (14 y-olds) or FJ and erosion, but cross-sectional data for the baseline (12 y-old) indicating an association between FJ and erosion is included. Authors suggest the lack of association may result from a decrease in FJ consumption over the 2 y time period.
El Aidi et al. (25)	13–15 y, *N =* 572	Permanent	Mean (range) intake, 3.1 (0, 21.88) glasses/d 67.1% of children consumed >1 glass/wk, 10.5% of children consumed >7 glasses/wk	Association of FJ with incidence of erosion: OR = 0.94, *P =* 0.140 Association of FJ with progression of erosion: OR = 0.97, *P =* 0.582	n/a	No association of fruit drink with incidence or progression of erosion. Acid drinks, which included fruit drinks and tooth grinding together were significant for erosion.
**CARIES AND MINERALIZATION**						
Marshall et al. (26, 27)	5 y, *N =* 634–642	Deciduous	Median (25th, 75th percentiles): Total Events/d: 1.0 (0.5, 1.4) Exposure at: Meals: 0.4 (0.2, 0.8); Snacks: 0.4 (0.2, 0.8).	n/a	100% FJ median (25th, 75th percentiles) intake: Caries-free: 114 (56, 188) g/d; Caries present: 107 (62, 166) g/d Caries risk for low intake 100% FJ: OR (95% CI) = 0.57 (0.34, 0.97), (*P* <0.05) Caries risk for High vs. Low quartile for 100% FJ (OR, 95% CI) = 0.90 (0.44, 1.85)	Overall, results suggest 100% FJ and soda pop are fundamentally different with respect to caries risk, with soda pop increasing caries risk more than 100% FJ.Timing of consumption has minimal impact on risk; no significant relationship with meal vs. snack intake overall.
Lim et al. (28)	5–7 y, *N =* 369	Deciduous	Baseline mean consumption/d (%total): 100% FJ: 588.6 mL (40%); Milk: 214.1 mL (22%); Soft drinks: 142.4 (10%) Results reported as High vs. Low consumers	n/a	Compared to High-High milk-FJ group: Low-High soft drink group >75x and >2.67 risk of d2mfs and f, respectively High-High soft drink group similar risk of new f surfaces, but not new d2mfs	Data for FJ confounded by inclusion with milk in the reported results. Authors concluded that those who increase their consumption of soft drinks compared to milk and 100% FJ are at higher risk of developing dental caries.
Chankanka et al. (29)	5 y, 9 y, 13 y, *N =* 156	Age 5, deciduous; Age 9, mixed; Age 13, permanent	Occasions/d (% N) Age 5: Low: <0.44 (25%); Medium: 0.44–1.33 (50.6%); High: >1.33 (24.4%) Age 9: Low: <0.11 (20.5%); Medium: 0.11–0.78 (58.3%); High: >0.78 (21.2%) Age 5: Low: <0.03 (22.4%); Medium: 0.03–0.57 (50%); High: >0.57 (27.6%)	n/a	Non-cavitated caries (mean): Age 5, 24.15%; Age 9, 39.10%; Age 13, 35.90%. The only statistically significant dietary variable (*P* <0.15) was frequency of 100% juice exposure. New non-cavitated caries surfaces	Greater frequency of 100% juice exposure was significantly associated with fewer non-cavitated and acavitated caries surfaces. Authors concluded that less frequent 100% juice exposure might be associated with higher exposure to several other cariogenic beverages.
					(mean): Age 5, 0.56; Age 9, 0.99; Age 13, 0.87. Frequency of 100% juice and powdered beverages were significant.	
Ghazal et al. (20)	2 y, *N =* 86; 3 y, *N =* 84; 4 y, *N =* 73	Deciduous	At baseline, 98, 92, and 99% of children consumed milk/infant formula, 100% FJ, and water, respectively	n/a	Total (% children with d, m, f > 0) at baseline, 1.1%. Total (% children with dmf>0) at 2, 3, and 4 y were 12.8, 39.3, and 65.8%, respectively. Children who consumed 100% juice 1x/d or more had ~ 60% lower odds of developing dental caries at 3 y compared to those who consumed 100% juice less frequently/d (*P =* 0.049).	Negative association between increased daily frequency of consumption of 100% juice and early childhood caries incidence might be due in part to the negative correlation between daily frequency of consumption of 100% juice and daily frequency of consumption of sugar-added beverages

*CI, confidence interval; d, day; dmf, decayed, missing, filled; FJ, fruit juice; IR, incidence rate; N, subject number; n/a, not applicable; OR, odds ratio; RR, risk ratio; y, year*.

The three cohort studies on erosion and juice included one study (23) in children with deciduous teeth (*N* = 355, age 5 y), and two studies (24, 25) in adolescents with permanent teeth (*N* = 2,325, age 12–15 y), with follow-up of 2–5 y. All three studies found no association between juice and tooth erosion, although only two (24, 25) of the three provide quantitative data. One of the studies also included a cross-sectional assessment of the baseline data for the cohorts, which indicated an association; however, the 2-year follow-up data from cohorts did not indicate an association (24). The studies on erosion utilized similar evaluation approaches, with two including oral examinations by trained and calibrated examiners (24, 25), and one utilizing tooth impressions that were assessed by two independent examiners (23). The approach to assessing fruit juice exposure, however, varied considerably, with only one of the three reports clearly differentiating fruit drinks from fruit juice (23). The other reports mentioned juice, but did not clearly indicate that only 100% fruit juice was included (24, 25). The approach to analysis also varied, with one not including confounders or adjusting for multiple comparisons (23), while the other two used an approach for multivariate analysis and/or addressed confounding, albeit different factors were included (24, 25). One of the studies indicated some funding from industry (25), and the other two did not report funding source (23, 24).

The studies on caries and juice were more varied in the population studies, but similar to the erosion studies, only included children and adolescents. Overall, the five studies on caries and/or mineralization represented data from 1,232 to 1,253 children ranging in age from 2 to 13 y, which included children with deciduous and permanent teeth, with follow-up of 2–13 y (20, 26–29). These studies reported either no association or an inverse association between intakes of 100% fruit juice and the incidence of dental caries.

### Randomized Controlled Trials

Nine RCTs that assessed the effect of 100% fruit juice on an aspect of dental health were identified, with six of these on fruit juice and erosion, and three on fruit juice and caries. The RCTs on erosion and fruit juice are summarized in Table 3, and those on dental caries and fruit juice outlined in Table 4.

**Table 3:** Description of randomized controlled trials on 100% fruit juice and tooth erosion.

**References**	**Country**	**Trial design**	**Duration**	**Subjects**	**Age**	**Sex**	**Erosion assessment**	Control/ comparator^*^	**100% Fruit juice intervention**	Results^*^
Dever et al. (21)	New Zealand	Crossover	12 d (10 d exposure, 2 rest) per each test	*N =* 8 (5 completed) Dentistry School staff	nr	nr	*In situ*, intra-oral appliance, plaque allowed to accumulate; Knoop microhardness, X-ray	200 mL swished and expectorated Sweetened, strawberry-flavored milk drink	200 mL swished and expectorated Apple-based orange-and-mango pure FJ	Knoop microhardness change after 5 d: Milk: −0.2 mcM (SD 1.8) mcM, ns from baseline. 100% FJ: −10.8 mcM (SD 5.0) mcM, *P* < 0.01
West et al. (30)	UK	Crossover Randomized Single-blind	15 working d; 2.5 d washout	*N =* 10 Adults, healthy and dentally fit	Mean: 24 y (20–30 y)	60% F	*In situ* intra-oral appliance, measured with surfometer, Wallace Micro- Indentation Tester	250 mL sipped over 10 min 4x/d: Water	250 mL sipped over 10 min 4x/d: OJ (pH, 3.74; 0.87% w/v titratable acidity)	Change in surface enamel loss at 15 d (between group P < 0.002): Control: +0.018 (SD 0.04) mcM 100% FJ: −2.69 (SD 0.49) mcM Surface microhardness mean (SD) unexposed vs. exposed Wallace Hardness Unit: Control: 49.9 (4.1) vs. 50.1 (4.3), *P =* 0.82 100% FJ: 48.3 (3.4) vs. 50.5 (3.5), *P =* 0.002 Exposed-unexposed difference greater for OJ than water, *P* = 0.049
Hughes et al. (31)	UK	Crossover Randomized Single-blind	15 working d; 2.5 d washout	*N =* 12 Adults, healthy and dentally fit	Mean: 27.5 y (20-34 y)	83% F	*In situ* intra-oral appliance, measured with surfometer	250 mL sipped over 10 min 4x/d: Water (pH 7.8, 29 ppm Ca)	250 mL sipped over 10 min 4x/d OJ (Sainsbury, UK; pH 3.8, 70 ppm Ca)	Mean (SD) enamel at 0, 5, 10, 15 d: Water: −0.19 (0.05), −0.07 (0.10), −0.02 (0.19), 0.00 (0.16) mcM; OJ:- 0.17 (0.09), −0.74 (0.33), −1.29 (0.38), −2.37 (1.11) mcM OJ significantly greater enamel loss than water at all timepoints (*P =* 0.001)
West et al. (32)	UK	Crossover Randomized Single-blind	15 working d; 2.5 d washout	*N =* 12 Adults, healthy and dentally fit	Mean: 26.7 y (20-39 y)	92% F	*In situ* intra-oral appliance, measured with surfometer	250 mL sipped over 10 min 4x/d: Water (pH 7.8, 29 ppm Ca)	250 mL sipped over 10 min 4x/d OJ (Del Monte, UK; pH 3.95, 167 ppm Ca)	Mean (SD) enamel at 3, 6, 9, 12, 15 d: Water: −0.05 (0.09), −0.08 (0.20), −0.10 (0.14),−0.10 (0.11), −0.05 (0.15) mcM; OJ: −0.52 (0.26),– 0.82 (0.46), −1.18 (0.76), −1.35 (0.52), −1.70 (0.70) mcM OJ significantly greater enamel loss than water at all timepoints *P* < 0.001
Hughes et al. (33)	UK	Crossover Randomized Single-blind	12 d (10 working d exposure, 2 d rest); 2.5 d washout	*N =* 12 Adults with a high level of oral hygiene and no excessive tooth wear	Mean: 22.5 y (22–42 y)	nr	*In situ* intra-oral appliance, scanning force microscopy, softened enamel by ultrasound Eisenberg removal	250 mL sipped over 10 min 4x/d at 0900, 1100, 1400, 1500 h: water (7.83 pH, 9.9 mg/L Ca)	250 mL sipped over 10 min 4x/d at 0900, 1100, 1400, 1500 h: OJ (3.3) pH, 120 mg/L Ca	Mean (SD) erosion depths at 2, 5, 10 d: Water: 0.10 (0.12), 0.05 (0.12), 0.18 (0.13) mcM; OJ: 0.19 (0.25), 0.53 (0.60), 2.03 (2.26) mcM; Mean (SD) softening at days 5 and 10: Water: 0.13 (0.48), 0.15 (0.65); OJ: 0.69 (1.06), 2.02 (2.90)
Finke et al. (34)	UK	Crossover Randomized Single-blind	5 d	*N =* 12 Adults	18–60 y	50% F	*In situ*, intra-oral appliance, Atomic force microscopy	4 ×250 mL sipped over 10 min: Mineral water (Danone)	4 ×250 mL sipped over 10 min: OJ (Sunny Delight™, Gerber)	Mean (SD) pellicle layer thickness at 2, 5 d: Water 120 (70) and 180 (40) nM; OJ: 170 (90) and 200 (50) nM Significantly greater pellicle layer with OJ compared to water.

* *Only results for the 100% FJ and water or no intervention controls are shown. Data on juice drinks and other beverages are not in provided herein*.

*Ca, calcium; d, day; F, female; FJ, fruit juice; h, hour; L, liter; mcM, micrometer; mg, milligram; min, minute; mL. milliliter; OJ, orange juice; N, subject number; nM, nanometer; ns, not significant; ppm, parts per million; SD, standard deviation; w/v, weight/volume; y, year*.

**Table 4:** Description of randomized controlled trials on 100% fruit juice and caries.

**References**	**Country**	**Trial design**	**Duration**	**Subjects**	**Age**	**Sex**	**Caries assessment**	Control/ comparator^*^	**100% Fruit juice intervention**	**Results**
Tenovuo and Rekola (35)	Finland	Crossover	2-wk test periods; 1-wk washout	*N =* 39 generally healthy, dentally fit dental students	18–23 y	46% F	Assessed salivary flow rate (mL/min) by time needed for collection of stimulated (8 mL) and unstimulated (2 mL) whole saliva, and plaque indices	*Ad libitum* at least 5x/d non-fluoridated water	*Ad libitum* at least 5.62 (SD 1.28)x/d: OJ (pH, 3.5; citric acid, 800 mg/100 mL; vit C, 40 mg/mL; total sugars,10 g/100 mL; caffeine, 0; fluoride, 0.009 mg/100 mL).	Immediately after consumption and at 10 min, OJ salivary flow and pH were greater than water (*P* < 0.05), with resolution occurring by 30 min (ns), but no difference was noted in this response after chronic (2-wk) consumption of the beverages. Oral fluid concentrations of calcium, phosphate, lactate, and fluoride, and plague lactate were not different.
Jensen et al. (36)	USA	Crossover	2 wk per test	*N =* 15 healthy adults	Mean: 36 y (F) and 38 y (M)	47% F	Intra-oral appliance with caries-like lesions and acid-resistant varnish, remineralization assessed by microradiography	No snacks	8 oz consumed 3x/d: OJ (Kemps, Marigold Foods) 8 oz. AJ (Speas Farms, Sundor Brands)	OJ resulted in enamel demineralization, but remineralization in dentin. AJ showed demineralization in both enamel and dentin. Authors in abstract indicate OJ led to remineralization in enamel, whereas AJ results in caries progression in enamel and dentin.
Issa et al. (37)	UK	Crossover	10-d test period; 7-d washout	*N =* 10 adults, dentally fit with normal salivary function	Mean: 37.2 y	40% F	Intra-oral appliance allowing for plaque development, mineralization of white spot lesion measured with microradiography	35 g over 7 events/d Positive control: 10% sucrose Negative control: 10% sorbitol	35 g juiced over 7 events/d Apples (13.72% total sugars); Oranges (6.29% total sugars); Grapes (14.97% total sugars); Tomatoes (2.85% total sugars)	Baseline DMFS 28.8, Significant demineralization with juices of tomatoes, apples, oranges, and grapes (*P* < 0.05). No difference between these fruits and the positive control, or when consumed as solid food or as juice. No significant demineralization was found with sorbitol.

* *Only results for the 100% FJ and water or no intervention controls are shown. Data on juice drinks and other beverages are not in provided herein*.

*AJ, apple juice; d, day; DMFS, decayed, missing, filled surfaces; F, female; h, hour; L, liter; M, male; mg, milligram; min, minute; mL. milliliter; N, subject number; ns, not significant; OJ, orange juice; oz, ounce; vit, vitamin; wk, week; SD, standard deviation; y, year*.

Of the six RCTs which addressed fruit juice and erosion, one compared fruit juice to milk (21), while the other five compared fruit juice to water (30–34). All but one (21) of these studies used orange juice as the 100% fruit juice, and several of these included orange juice as a positive control. All studies were crossover designs, varying from 5 to 15 days of beverage exposure, and small, having only 5–12 evaluable subjects. The subjects in the studies were adults. Five studies indicated using a randomized single-blind approach, with no detail provided by one study (21). Four (30–33) of the six studies measured enamel loss, one measured thickness of the pellicle (34), which is a protein film on the surface of the enamel, and one study assessed the hardness of the enamel and dentin (21). Each study was conducted as an *in situ* analysis using an intra-oral device developed with a machined enamel slab, and the device was cleaned of plaque every day. Therefore, these data did not assess enamel loss in the presence of normally accumulated plaque. All RCTs were rated as having some concerns using the RoB 2 tool due to possible presence of bias arising from the randomization process and effect of assignment to intervention. Meanwhile, the Dever et al. (21) study was rated as having critical risk of bias using the ROBINS-I assessment tool. Concerns of bias for this study were mostly due to confounding, classification of interventions, and missing data.

All six RCTs on tooth erosion and fruit juice (21, 30–34) showed a positive relationship between 100% juice consumption and decreased microhardness, surface enamel loss, erosion depth, enamel softening, and increased pellicle layer. However, the data on erosion and fruit juice are limited, with the data comparing fruit juice to water or no intervention. In addition, four (30–33) of the six studies were conducted by the same research team. The other two studies either indicated co-funding by industry, or did not indicate a funding source (Table 5). Therefore, independent data on fruit juice and erosion were not found.

**Table 5:** Risk of bias assessment of randomized controlled studies on 100% fruit juice and dental health.

**References**	**Randomized**	**Sequence generation**	**Allocation concealment**	**Blinding**	**Analysis population**	**Analysis approach**	**Incomplete data**	**Dropouts (%)**	**Funding**
**EROSION**									
Dever et al. (21)	nr	nr	nr	nr	Completers	Paired *t*-test, adjusted for unequal variances. No other confounder adjustments reported	nr	37.5%	nr
West et al. (30)	Yes	nr	nr	Single	ITT	Paired non-parametric Wilcoxon, No adjustments	n/a	0%	Industry
Hughes et al. (31)	Yes, balanced for residual effects	nr	nr	Single	ITT	Paired *t*-test No reported adjustments	n/a	0%	nr
West et al. (32)	Yes	nr	nr	Single	ITT	Paired *t*-test No reported adjustments	n/a	0%	nr
Hughes et al. (33)	Yes	nr	nr	Single	ITT	ANOVA paired *t-*test between beverages subjects, period, treatment used in statistical models	n/a	0%	nr
Finke et al. (34)	Yes, balanced for 1st order crossover effects	nr	nr	Single	nr	3-way ANOVA and multiple range test (Fisher's LSD) drinks, exposure time, volunteers used in statistical models	nr	nr	Industry & Government Grant Funded
**CARIES AND MINERALIZATION**									
Tenovuo and Rekola (35)	nr	nr	nr	nr	ITT	Student's *t*-test	n/a	0%	Industry
Jensen et al. (36)	Yes	nr	nr	nr	nr	ANOVA followed by Tukey's protected *t*-test No reported adjustments	nr	20%	nr
Issa et al. (37)	Partial at lab site only	nr	nr	Single at lab site	nr	Paired *t*-test, 1-way ANOVA, *post-hoc* Tukey's test for multiple comparisons No reported adjustments	nr	nr	Industry

*d, day; ITT, intent-to-treat; n/a, not applicable; nr, not reported*.

Of the three RCTs which assessed caries or mineralization/demineralization, two (35, 36) assessed orange juice, with one of these also including apple juice (36), while the other study compared a number of fruits, including orange, grapes, and apples consumed as freshly juiced beverages or as whole fruits (37). Although these studies tended to have similar designs (e.g., crossover, duration 10–14 days, dentally fit adults), they varied substantially with respect to analyses. One study assessed salivary and plaque indices (35), while the other two utilized an *in situ* approach with intra-oral appliances and reported on aspects of mineralization (36, 37). Overall, there is limited RCT data for fruit juice and caries, and the study designs were quite varied. One study suggested orange juice did not lead to pro-cariogenic changes in salivary and plaque parameters (35), and the two studies on mineralization indicated demineralization occurs with fruit juices (36, 37), although one of these reported mixed results for orange juice (36). Two of the studies indicated funding from industry (35, 37), and one did not report a funding source (36). Only one of the studies clearly indicated randomization (36), while one indicated the study design did not allow for full randomization (37), and the other study did not report randomization (35). There was some suggestion that not all subjects were included in the respective analyses in two of the studies (36, 37).

## Discussion

Sugar-containing foods and beverages have been a focus of public policy recommendations related to dental health, principally dental caries and tooth erosion (6, 38). However, these reports have combined data from multiple sources of foods and have not specifically addressed 100% fruit juices. Instead, conclusions on 100% fruit juice have most often been developed from data on sugar-sweetened beverages and/or foods that have either combined the 100% fruit juice data with data on other foods or beverages, or from data which did not include 100% fruit juice. This report provides the results of intervention and prospective cohort studies that have specifically addressed the effects of 100% fruit juice on outcomes or markers of dental health. Results from prospective studies in adolescents and children indicate that 100% fruit juice consumption is not associated with incidence of dental caries and tooth erosion. However, intervention studies in adults suggest that 100% fruit juice could contribute to increased tooth erosion or negative effects on markers of dental caries, although these studies primarily utilized *in-situ* intra-oral enamel appliances.

To the best of our knowledge, only one systematic review has assessed 100% fruit juice and dental health as its primary focus, which included five cross-sectional studies with two prospective cohort studies that assessed erosion as a sub-analysis only, and reported a possible association with 100% fruit juice (OR = 1.20; 95% CI 0.02–1.42, *p* = 0.03), although the data indicated high heterogeneity (*I*^2^ = 74.6%, *p* = 0.001) (14). Most notably, the two prospective cohorts in that analysis reported no association, indicating the positive association was found only in the cross-sectional data and was not supported by prospective studies. The present review did not include cross-sectional studies and identified one additional prospective cohort that also reported no association between juice intake and tooth erosion (23). Although the present review did not include case-controls, two case-controls studies on tooth erosion and 100% fruit juice were identified during the search process and results were mixed. One study reported no significant relationship between fruit juice consumption and tooth wear in children in Liverpool, UK (*N* = 60, 15 y) (39), while another found a significant association between the duration of intake of orange juice and tooth wear in Malaysian children (*N* = 576, 16 y) (40). However, neither of these case-control studies clearly described the fruit juice as being 100% fruit juice.

Meanwhile, the five prospective cohort studies on 100% juice intakes and caries incidence reported herein found either no association or an inverse association. The search process also identified three case-control studies on caries and fruit juice and results were also mixed, with two reports of a positive association between fruit juices and root caries in adults (*N* = 275) and early childhood caries (*N* = 119) (41, 42) and one report of an inverse relationship between fruit juice consumption and early childhood caries in preschool children in Egypt (*N* = 60) (43). However, similar to those on tooth erosion, none of these case-control studies clearly described the fruit juice as being 100% fruit juice.

With respect to RCT data, three RCTs were identified that assessed caries or mineralization/demineralization and six RCTs reported on 100% fruit juice and erosion (21, 30–34). All six studies reported a positive relationship between 100% fruit juice consumption and erosion or markers for cavities (e.g., decreased microhardness, increased surface enamel loss, increased erosion depth, greater enamel softening, and/or increased pellicle layer). Additionally, studies on caries and fruit juice showed increased demineralization of enamel slabs despite increased acute saliva production, which is considered a protective factor. With the exception of one study that assessed saliva production, all the clinical trials identified in this review employed *in situ* intra-oral appliances, whereby enamel samples were placed in dental appliances worn by subjects who were then instructed to consume the test beverages generally throughout the day. This allowed changes to be observed within the context of the subjects' normal diet and dental hygiene, although the appliances were cleaned of plaque daily. The studies were generally short (≤ 15 days) and small (all but one study included ≤ 12 subjects). In addition, the frequency of juice consumption in these studies was often greater compared to normal conditions of consumption, with the intake of the fruit juice and/or number or events several times higher (generally>750 mL or at least 4 events per day) than average intakes [<250 mL per day (5)]. Therefore, the clinical studies do not represent the normal intake conditions, and instead, were designed to detect potential changes using aggressive methods.

Studies on the effect of diet on dental health are challenging. For example, the methodology for assessing tooth erosion and caries is heterogeneous, likely because techniques for assessing erosive damage or demineralization are heterogeneous. While there are several techniques that have found acceptance in published studies, none have achieved scientific consensus or certification by an authoritative body (44, 45). Another limitation of the clinical studies of erosion and dental caries is the challenge of blinding subjects to the intervention. The identities of different juices are virtually impossible to conceal due to distinct and identifiable color and sensory differences. The enamel slabs may be more susceptible to erosion as they are either human enamel slabs which can have reduced hardness because they are taken from below the outer surface of extracted human teeth, where the enamel is the hardest (30); or bovine enamel which is more porous than human enamel (46). In addition, the number of available subjects has generally been small, often recruited from the students and faculty of dental schools. Where multiple products have been tested, the order of testing of products assigned to individuals or groups of subjects can be randomized, which has been the case for crossover studies of multiple products.

### Limitations

All prospective cohort studies were conducted in children and adolescents while RCTs were only in adults, and the differences in populations between the two types of studies may contribute to the observed differential outcomes. The prospective studies also reflect more normal intakes of the juices and dental hygiene practices, compared with the clinical studies, which utilized high intake and consumption approaches. For example, prospective cohort studies used either food frequency questionnaires or diet records to assess habitual intakes of fruit juice, which better reflects normal consumption of fruit juice relative to the identified clinical studies, and thus, the progression of tooth erosion or caries. Furthermore, the prospective cohort studies also have the advantage of assessments of outcomes, i.e., erosion or caries, rather than the surrogate markers found in the clinical studies. However, as with other observational studies, prospective cohort studies strongly aid in studying causal associations but cannot distinguish true causality.

The studies included in this review may not represent all prospective cohort and intervention studies on 100% fruit juice and dental health because studies not published in English were excluded and those not cited in the databases used may have been missed. However, effort was made during this review to hand search all references in selected reviews and reports. In addition, this review excludes single meal studies and those in diseased populations, and thus does not contribute information about the acute effects of 100% fruit juice on dental health and in diseased individuals. Finally, observational studies other than prospective cohorts (e.g., cross-sectional, case-control, case series) were not included as these are generally not considered in many evidence-based reviews due to the likelihood of high bias.

### Conclusions

Products meeting the definition of 100% fruit juice comprise a distinct class of beverage and have been identified as candidates for limited consumption by the general population by virtue of their content of sugars and relatively low pH. The rationale for such a recommendation stemmed from the perception that the consumption of fruit juices might contribute to tooth erosion, dental caries, or both. However, the existing evidence on 100% fruit juice intake and dental caries or tooth erosion are not conclusive. Prospective cohort studies in children and adolescents found no association between 100% fruit juice intake and tooth erosion and no association or inverse association between 100% fruit juice intake dental caries, whereas RCTs in adults suggest that 100% fruit juice could contribute to tooth erosion and dental caries. Although the RCT is the gold-standard for demonstrating cause-and-effect, the RCTs on 100% fruit juice and dental health have employed conditions that were extreme for amounts and exposures relative to normal intakes of 100% fruit juices. In addition, other methodological concerns, such as using more susceptible enamel slabs for experiments, could have contributed to the differential results found with the RCTs. Further, due to the challenges in clinical studies on dental health outcomes, particularly in vulnerable populations such as children, the development of consensus study techniques which more accurately reflect the dynamics of healthy teeth is also warranted. Therefore, well-designed, larger intervention studies on 100% fruit juice and dental health outcomes that implement consensus study techniques which more accurately reflect the dynamics of healthy teeth in both adults and children are needed for policy-making and clinical recommendations on intake of 100% fruit juice.

## Author Contributions

DL and EM were responsible for the systematic search, screening, and data extraction. DL and MK prepared the original draft manuscript. All authors reviewed and edited the manuscript and approved the final version.

### Conflict of Interest Statement

DL and EM received partial funding from Juice Products Associations to conduct this systematic review. MK received a consulting fee from the Juice Products Association for his contributions to this manuscript. DL, EM, and MK provide consulting services in nutrition science to industry associations, companies and other organizations. Juice Product Association was involved in the conceptualization of this project, but was not involved in the screening, data extraction, interpretation, and writing of this manuscript.
