# Supplementary material for: 100% Fruit Juice and Dental Health: A Systematic Review of the Literature
Source: Front Public Health. 2019 Jul 12;7:190. doi: 10.3389/fpubh.2019.00190 (PMC6640211; doi:10.3389/fpubh.2019.00190)
Supplement: Supplementary file 1 [file Table_1.docx]

Supplementary Material

**Supplementary Table S1.**  Search Strategy

| **Database** | **Search Terms and Limits** |
| --- | --- |
| MEDLINE/ PubMed | ***TOPIC***: ((Juice or juices)  ***AND*** (Fruit or apple or grape or orange or lemon or lime or grapefruit or cranberry or raspberry or cherry or apricot or banana or kiwi or guava or tomato or blueberry or melon or pumpkin or blackberry or *berry) OR Sugar-sweetened beverages OR sugar-sweetened beverage)  ***AND*** (Oral or teeth or tooth or dental or caries or cavity)  ***AND*** (Human)  ***AND*** English |
| EMBASE | ***TOPIC:*** (('juice'/exp OR juice OR juices) AND ('fruit'/exp OR fruit OR 'apple'/exp OR apple OR 'grape'/exp OR grape OR 'orange'/exp OR orange OR 'lemon'/exp OR lemon OR 'lime'/exp OR lime OR 'grapefruit'/exp OR grapefruit OR 'cranberry'/exp OR cranberry OR 'raspberry'/exp OR raspberry OR 'cherry'/exp OR cherry OR 'apricot'/exp OR apricot OR 'banana'/exp OR banana OR kiwi OR 'guava'/exp OR guava OR 'tomato'/exp OR tomato OR 'blueberry'/exp OR blueberry OR 'melon'/exp OR melon OR 'pumpkin'/exp OR pumpkin OR 'blackberry'/exp OR blackberry) OR (('sugar sweetened'  ***AND*** ('beverages'/exp OR beverages) OR 'sugar sweetened')  ***AND*** ('beverage'/exp OR beverage))) AND (oral OR 'teeth'/exp OR teeth OR 'tooth'/exp OR tooth OR 'caries'/exp OR caries OR cavity OR 'dental'/exp OR dental)  ***AND*** ([humans]/lim)  ***AND*** ([english]/lim) |
| Web of Science (WOS) | **TOPIC:** (Juice or juices) AND TOPIC: (Fruit or apple or grape or orange or lemon or lime or grapefruit or cranberry or raspberry or cherry or apricot or banana or kiwi or guava or tomato or blueberry or melon or pumpkin or blackberry) OR (Sugar-sweetened beverages OR sugar-sweetened beverage)  ***AND*** (Oral or teeth or tooth or dental or caries or cavity)  ***INDEXES***= SCI-EXPANDED, SSCI, A&HCI, ESCI Timespan=All years |
| Cochrane | Search Category switched from (title, abstract, keyword) to (search all text) for:  "fruit juice OR fruit juices"  ***AND*** "dental OR teeth OR tooth OR caries OR cavity" |

**Supplementary Table 2a.** Excluded Acute Intervention Studies (n=15)

| **Citation** | **Title** | **Reason for Exclusion** |
| --- | --- | --- |
| Tenuta et al., 2015.(1) | Titratable acidity of beverages influences salivary pH recovery | Acute intervention |
| Mahajan et al., 2014.(2) | Effect of commonly consumed sugar containing and sugar free fruit drinks on the hydrogen ion modulation of human dental plaque | Acute intervention |
| Scaramucci et al., 2012.(3) | *In situ* evaluation of the erosive potential of orange juice modified by food additives | Acute intervention |
| Lussi et al., 2012.(4) | Clinical study monitoring the pH on tooth surfaces in patients with and without erosion | Acute intervention |
| Preethi et al., 2010.(5) | The effect of four fruit juices on the pH of dental plaque - a four period cross-over study | Acute intervention |
| Hannig et al., 2009.(6) | Polyphenolic beverages reduce initial bacterial adherence to enamel *in situ* | Acute intervention |
| Hannig et al., 2009.(7) | The effect of acidic beverages on the ultrastructure of the acquired pellicle--an *in situ* study. | Acute intervention |
| Azrak et al., 2008.(8) | Course of Changes in Salivary pH-Values after Intake of Different Beverages in Young Children | Acute intervention |
| Johansson et al., 2007.(9) | Effect of soft drinks on proximal plaque pH at normal and low salivary secretion rates | Acute intervention |
| Banan & Hedge, 2005.(10) | Plaque and salivary pH changes after consumption of fresh fruit juices | Acute intervention |
| Azrak et al., 2003.(11) | Reduction of the pH-values of whole saliva after the intake of apple juice containing beverages in children and adults | Acute intervention |
| Hussein et al., 1996.(12) | A comparison of the effects of some extrinsic and intrinsic sugars on dental plaque pH | Acute intervention |
| Vassilakos et al, 1990.(13) | Oral electrochemical action after soft drink rinsing and consumption of sweets | Acute intervention |
| Birkhead, 1984.(14) | Sugar content, acidity and effect on plaque pH of fruit juices, fruit drinks, carbonated beverages and sport drinks | Acute intervention |
| Naval et al. 2013.(15) | The effects of beverages on plaque acidogenicity after a sugary challenge. | Acute intervention |

**Supplementary Table 2b.** Other Excluded Studies from Full-text Search

| **Citation** | **Title** | **Reason for Exclusion** |
| --- | --- | --- |
| Dehghan et al., 2017.(16) | Neutralizing salivary pH by mouthwashes after an acidic challenge | Not a 100% FJ intervention. FJ used as a challenge for testing other interventions. |
| Turssi et al., 2015.(17) | Kinetics of salivary pH after acidic beverage intake by patients undergoing orthodontic treatment | Not a 100% FJ intervention. FJ used as a challenge for testing other interventions. |
| Olley et al., 2015.(18) | Effects of dentifrices on subsurface dentin tubule occlusion: an *in situ* study | Not a 100% FJ intervention. FJ used as a challenge for testing other interventions. |
| Hooper et al., 2014.(19) | A randomized *in situ* trial, measuring the anti-erosive properties of a stannous-containing sodium fluoride dentifrice compared with a sodium fluoride/potassium nitrate dentifrice | Not a 100% FJ intervention. FJ used as a challenge for testing other interventions. |
| Hara et al., 2014.(20) | Novel in-situ longitudinal model for the study of dentifrices on dental erosion-abrasion | Not a 100% FJ intervention. FJ used as a challenge for testing other interventions. |
| Wang et al., 2013.(21) | Dentin tubule occlusion and erosion protection effects of dentifrice containing bioadhesive PVM/MA copolymers | Not a 100% FJ intervention. FJ used as a challenge for testing other interventions. |
| Olley et al., 2012.(22) | An *in situ* study investigating dentine tubule occlusion of dentifrices following acid challenge. | Not a 100% FJ intervention. FJ used as a challenge for testing other interventions. |
| Hooper et al., 2007.(23) | The protective effects of toothpaste against erosion by orange juice: studies *in situ* and *in vitro* | Not a 100% FJ intervention. FJ used as a challenge for testing other interventions. |
| Zero et al., 2006.(24) | Evaluation of a desensitizing test dentifrice using an *in situ* erosion remineralization model | Not a 100% FJ intervention. FJ used as a challenge for testing other interventions. |
| Hara et al., 2006.(25) | Protective effect of the dental pellicle against erosive challenges *in situ* | Not a 100% FJ intervention. FJ used as a challenge for testing other interventions. |
| Hooper et al., 2003.(26) | Investigation of erosion and abrasion on enamel and dentine: a model *in situ* using toothpastes of different abrasivity | Not a 100% FJ intervention. FJ used as a challenge for testing other interventions. |
| Pontefract et al., 2001.(27) | The erosive effects of some mouthrinses on enamel. A study *in situ* | Not a 100% FJ intervention. FJ used as a challenge for testing other interventions. |
| Amaechi et al. 2003.(28) | Influence of abrasion in clinical manifestation of human dental erosion | Not a 100% FJ intervention. FJ used as a challenge for testing other interventions. |
| Mullan et al. 2018.(29) | An in-situ pilot study to investigate the native clinical resistance of enamel to erosion | Not a 100% FJ intervention. FJ used as a challenge for testing other interventions. |
| Benson et al. 2014.(30) | Occurrence of habituation during repeated food exposure via the olfactory and gustatory systems | No dental health outcome |
| Chapple et al. 2010. (31) | The effect of supplementation with adjunctive whole fruit/vegetable/berry concentrate on outcomes of periodontal therapy | Abstract, not full report. |
| Bassiouny et al. 2008.(32) | Topographic and radiographic profile assessment of dental erosion. Part II: effect of citrus fruit juices on human dentition. | In vitro |
| Altun et al. 2015.(33) | The erosive effects of honey, molasses and orange juice on the primary teeth of children | In vitro |
| Brusius et al., (2018) (34) | Dental erosion among South Brazilian adolescents: A 2.5-year longitudinal study. Community dentistry and oral epidemiology | No 100% FJ |
| Josino Soares et al. 2014.(35) | Pitanga (Eugenia uniflora L.) fruit juice and two major constituents thereof exhibit anti-inflammatory properties in human gingival and oral gum epithelial cells. | No 100% FJ |
| Gupta et al. 2015.(36) | Effect of high-molecular-weight component of Cranberry on plaque and salivary Streptococcus mutans counts in children: an in vivo study. | No 100% FJ |
| Pak et al. 1994.(37) | Effect of added citrate or malate on calcium absorption from calcium-fortified orange juice. | No 100% FJ |
| Hooper et al. 2004.(38) | A comparison of enamel erosion by a new sports drink compared to two proprietary products: a controlled, crossover study in situ | No 100% FJ |
| Ismail et al. 2009(39) | Predictors of dental caries progression in primary teeth | No 100% FJ |
| Gedalia et al. 1981.(40) | Effect of fluoridated citrus beverage on dental caries and on fluoride concentration in the surface enamel of children's teeth | No 100% FJ |
| Arnadóttir et al. 2003.(41) | Dental erosion in Icelandic teenagers in relation to dietary and lifestyle factors. | No 100% FJ |
| Hohensinn et al. 2016.(42) | Sustaining elevated levels of nitrite in the oral cavity through consumption of nitrate-rich beetroot juice in young healthy adults reduces salivary pH. | No 100% FJ |
| Macpherson & Dawes. 1994.(43) | Distribution of sucrose around the mouth and its clearance after a sucrose mouthrinse or consumption of three different foods. | No 100% FJ |
| Parkar et al. 2017.(44) | Consumption of apple-boysenberry beverage decreases salivary Actinomyces naeslundii and their adhesion in a multi-species biofilm model. | No 100% FJ |
| Zare Javid et al. 2018.(45) | Impact of Cranberry Juice Enriched with Omega-3 Fatty Acids Adjunct with Nonsurgical Periodontal Treatment on Metabolic Control and Periodontal Status in Type 2 Patients with Diabetes with Periodontal Disease | No 100% FJ |
| Toumba & Duggal. 1999.(46) | Effect on plaque pH of fruit drinks with reduced carbohydrate content. | No 100% FJ |
| Tahmassebi & Duggal. 1998.(47) | The effect of different methods of drinking on the pH of dental plaque in vivo. | No 100% FJ |
| Piangprach et al. 2009.(48) | The effect of salivary factors on dental erosion in various age groups and tooth surfaces. | No 100% FJ |
| Murugesh et al. 2015.(49) | Effect of yogurt and pH equivalent lemon juice on salivary flow rate in healthy volunteers - An experimental crossover study | No 100% FJ |
| Chapple et al. 2011.(50) | Adjunctive daily supplementation with encapsulated fruit, vegetable and berry juice powder concentrates and clinical periodontal outcomes: a double-blind RCT. | No 100% FJ |
| Levine et al. 2007.(51) | Dietary patterns, toothbrushing habits and caries experience of schoolchildren in West Yorkshire, England. | No 100% FJ |
| Watanabe et al. 2014.(52) | The influence of lifestyle on the incidence of dental caries among 3-year-old Japanese children. | No 100% FJ |
| Hung et al. 2005.(53) | The association between tooth loss and the self-reported intake of selected CVD-related nutrients and foods among US women. | No 100% FJ |
| Bernabe et al. 2014.(54) | Sugar-sweetened beverages and dental caries in adults: A 4-year prospective study | No 100% FJ |
| El Aidi et al. 2011.(55) | Multifactorial analysis of factors associated with the incidence and progression of erosive tooth wear | No 100% FJ |
| Aidi et al. 2011.(56) | Factors associated with the incidence of erosive wear in upper incisors and lower first molars: a longitudinal approach | No 100% FJ |
| West et al. 2003.(57) | Development of low erosive carbonated fruit drinks 2. Evaluation of an experimental carbonated blackcurrant drink compared to a conventional carbonated drink. | No 100% FJ |
| West et al. 2004.(58) | Modification of soft drinks with xanthan gum to minimize erosion: a study in situ | No 100% FJ |
| Hughes et al. 1999.(59) | Development and evaluation of a low erosive blackcurrant juice drink. 3. Final drink and concentrate, formulae comparisons in situ and overview of the concept. | No 100% FJ |
| Horswill et al. 2006.(60) | Effect of exercise and fluid consumption on salivary flow and pH. | No 100% FJ |
| Sauro et al. 2008.(61) | In situ enamel morphology evaluation after acidic soft drink consumption: protection factor of contemporary toothpaste. | No 100% FJ |
| Saeed & Al-Tinawi. 2010.(62) | Evaluation of acidity and total sugar content of children's popular beverages and their effect on plaque pH. | No 100% FJ |
| Rugg-Gunn et al. 1998.(63) | Comparison of erosion of dental enamel by four drinks using an intra-oral applicance. | No 100% FJ |
| Takayanagi et al. 1995.(64) | A modified device for long term sampling of parotid saliva in various experimental conditions. | No 100% FJ |
| Warren et al. 2008.(65) | A longitudinal study of dental caries risk among very young low SES children. | No 100% FJ |
| Weber-Gasparoni et al. 2013.(66) | An Effective Psychoeducational Intervention for Early Childhood Caries Prevention: Part II | No relevant outcome. |
| Livny A & Sgan-Cohen. 2007.(67) | A review of a community program aimed at preventing early childhood caries among Jerusalem infants--a brief communication. | No relevant outcome. |
| Ribeiro et al. 2017.(68) | In situ evaluation of color stability and hardness' decrease of resin-based composites | No relevant outcome. |
| Briso et al. 2016.(69) | An In Situ Study of the Influence of Staining Beverages on Color Alteration of Bleached Teeth | No relevant outcome. |
| Beck et al. 2017.(70) | Randomized Controlled Trial of a Clinic-Based Intervention to Promote Healthy Beverage Consumption Among Latino Children | No relevant outcome. |
| Marshall. 2013.(71) | Preventing dental caries associated with sugar-sweetened beverages. | Review |
| Bassiouny. 2014.(72) | Clinical observations of dental erosion associated with citrus diet and intake methods. | Review |
| Bassiouny. 2012.(73) | Effect of sweetening agents in acidic beverages on associated erosion lesions. | Review |
| Myklebust et al. 2003.(74) | Dental health behavior, gastroesophageal disorders and dietary habits among Norwegian recruits in 1990 and 1999. | Cross-section study |
| Kolker et al. 2008.(75) | Dental caries and dietary patterns in low-income African American children. | Cross-section study |
| Galon et al. 1982.(76) | Fluoridated citrus beverage for the prevention of dental caries: 3 years of flouride supply and 2 years cessation | Not full report, limited FJ description |
| Mannerberg. 1962.(77) | Effect of lemon juice on different types of tooth surface. A replica study in vivo. | Unable to get fulltext, and appears to be duplicate publication. |
| Dimitrova & Kukleva. 2008.(78) | [Model for early childhood caries risks]. | Article in Russian. |
| Mathew et al. 2018.(79) | Effect of Fruit Juices and Other Beverages on Loss of Tooth Structure | In vitro |
| Vieira et al. 2018.(80) | Milk Reverts the Effects of an Enamel Erosive but Healthy Diet | In vitro |

**References for supplementary tables**

1. Tenuta LM, Fernandez CE, Brandao AC, Cury JA. Titratable acidity of beverages influences salivary pH recovery. Braz Oral Res. (2015) 29.
2. Mahajan N, Kotwal B, Sachdev V, Rewal N, Gupta R, Goyal S. Effect of commonly consumed sugar containing and sugar free fruit drinks on the hydrogen ion modulation of human dental plaque. J Indian Soc Pedod Prev Dent. (2014) 32(1):26-32.
3. Scaramucci T, Sobral MA, Eckert GJ, Zero DT, Hara AT. In situ evaluation of the erosive potential of orange juice modified by food additives. Caries Res. (2012) 46(1):55-61.
4. Lussi A, von Salis-Marincek M, Ganss C, Hellwig E, Cheaib Z, Jaeggi T. Clinical study monitoring the pH on tooth surfaces in patients with and without erosion. Caries Res. (2012) 46(6):507-512.
5. Preethi BP, Maitreyee DS, Dodawad R. The effect of four fruit juices on the pH of dental plaque - a four period cross-over study. J Clin Diag Res. (2010) 4(3):2587 - 2593.
6. Hannig C, Sorg J, Spitzmuller B, Hannig M, Al-Ahmad A. Polyphenolic beverages reduce initial bacterial adherence to enamel in situ. J Dent. (2009) 37(7):560-566.
7. Hannig C, Berndt D, Hoth-Hannig W, Hannig M. The effect of acidic beverages on the ultrastructure of the acquired pellicle--an in situ study. Arch Oral Biol. (2009) 54(6):518-526.
8. Azrak B, Willershausen B, Meyer N, Callaway A. Course of changes in salivary pH-values after intake of different beverages in young children. Oral Health Prev Dent. (2008) 6(2):159-164.
9. Johansson AK, Lingstrom P, Birkhed D. Effect of soft drinks on proximal plaque pH at normal and low salivary secretion rates. Acta Odontol Scand. (2007) 65(6):352-356.
10. Banan LK, Hegde AM. Plaque and salivary pH changes after consumption of fresh fruit juices. J Clin Pediatr Dent. (2005) 30(1):9-13.
11. Azrak B, Callaway A, Knozinger S, Willershausen B. Reduction of the pH-values of whole saliva after the intake of apple juice containing beverages in children and adults. Oral Health Prev Dent. (2003) 1(3):229-236.
12. Hussein I, Pollard MA, Curzon ME. A comparison of the effects of some extrinsic and intrinsic sugars on dental plaque pH. Int J Paediatr Dent. (1996) 6(2):81-86.
13. Vassilakos N, Nilner K, Birkhed D. Oral electrochemical action after soft drink rinsing and consumption of sweets. Scand J Dent Res. (1990) 98(4):336-340.
14. Birkhed D. Sugar content, acidity and effect on plaque pH of fruit juices, fruit drinks, carbonated beverages and sport drinks. Caries Res. (1984) 18(2):120-127.
15. Naval S, Koerber A, Salzmann L, Punwani I, Johnson BR, Wu CD. The effects of beverages on plaque acidogenicity after a sugary challenge. J Am Dent Assoc. (2013) 144(7):815-822.
16. Dehghan M, Tantbirojn D, Kymer-Davis E, Stewart CW, Zhang YH, Versluis A et al. Neutralizing salivary pH by mouthwashes after an acidic challenge. J Investig Clin Dent. (2017) 8(2).
17. Turssi CP, Silva CS, Bridi EC, Amaral FL, Franca FM, Basting RT. Kinetics of salivary pH after acidic beverage intake by patients undergoing orthodontic treatment. Gen Dent. (2015) 63(3):26-30.
18. Olley RC, Moazzez R, Bartlett D. Effects of dentifrices on subsurface dentin tubule occlusion: an in situ study. Int J Prosthodont. (2015) 28(2):181-187.
19. Hooper S, Seong J, Macdonald E, Claydon N, Hellin N, Barker ML et al. A randomised in situ trial, measuring the anti-erosive properties of a stannous-containing sodium fluoride dentifrice compared with a sodium fluoride/potassium nitrate dentifrice. Int Dent J. (2014) 64 Suppl 1:35-42.
20. Hara AT, Barlow AP, Eckert GJ, Zero DT. Novel in-situ longitudinal model for the study of dentifrices on dental erosion-abrasion. Eur J Oral Sci. (2014) 122(2):161-167.
21. Wang Q, Kang Y, Barnes V, DeVizio W, Kashi A, Ren YF. Dentin tubule occlusion and erosion protection effects of dentifrice containing bioadhesive PVM/MA copolymers. Clin Oral Investig. (2013) 17(3):775-783.
22. Olley RC, Pilecki P, Hughes N, Jeffery P, Austin RS, Moazzez R et al. An in situ study investigating dentine tubule occlusion of dentifrices following acid challenge. J Dent. (2012) 40(7):585-593.
23. Hooper SM, Newcombe RG, Faller R, Eversole S, Addy M, West NX. The protective effects of toothpaste against erosion by orange juice: studies in situ and in vitro. J Dent. (2007) 35(6):476-481.
24. Zero DT, Hara AT, Kelly SA, Gonzalez-Cabezas C, Eckert GJ, Barlow AP et al. Evaluation of a desensitizing test dentifrice using an in situ erosion remineralization model. J Clin Dent. (2006) 17(4):112-116.
25. Hara AT, Ando M, Gonzalez-Cabezas C, Cury JA, Serra MC, Zero DT. Protective effect of the dental pellicle against erosive challenges in situ. J Dent Res. (2006) 85(7):612-616.
26. Hooper S, West NX, Pickles MJ, Joiner A, Newcombe RG, Addy M. Investigation of erosion and abrasion on enamel and dentine: a model in situ using toothpastes of different abrasivity. J Clin Periodontol. (2003) 30(9):802-808.
27. Pontefract H, Hughes J, Kemp K, Yates R, Newcombe RG, Addy M. The erosive effects of some mouthrinses on enamel. A study in situ. J Clin Periodontol. (2001) 28(4):319-324.
28. Amaechi BT, Higham SM, Edgar WM. Influence of abrasion in clinical manifestation of human dental erosion. J Oral Rehabil. (2003) 30(4):407-413.
29. Mullan F, Austin RS, Parkinson CR, Bartlett DW. An in-situ pilot study to investigate the native clinical resistance of enamel to erosion. J Dent. (2018) 70:124-128.
30. Benson K, Raynor HA. Occurrence of habituation during repeated food exposure via the olfactory and gustatory systems. Eat Behav. (2014) 15(2):331-333.
31. Chapple IL, Milward MR, Ling-Mountford N, deSpirt S, Weston P, Dallal GE et al. The effect of supplementation with adjunctive whole fruit/vegetable/berry concentrate on outcomes of periodontal therapy. Eur J Integrative Med. (2010) 2(4):213-213.
32. Bassiouny MA, Yang J, Kuroda S. Topographic and radiographic profile assessment of dental erosion. Part II: effect of citrus fruit juices on human dentition. Gen Dent. (2008) 56(2):136-143.
33. Altun C, Maden EA, Ucar BD, Polat GG. The erosive effects of honey, molasses and orange juice on the primary teeth of children. Pediatr Dent J. (2015) 25(2):50-53.
34. Brusius CD, Alves LS, Susin C, Maltz M. Dental erosion among South Brazilian adolescents: A 2.5-year longitudinal study. Community Dent Oral Epidemiol. (2018) 46(1):17-23.
35. Josino Soares D, Walker J, Pignitter M, Walker JM, Imboeck JM, Ehrnhoefer-Ressler MM et al. Pitanga (Eugenia uniflora L.) fruit juice and two major constituents thereof exhibit anti-inflammatory properties in human gingival and oral gum epithelial cells. Food Funct. (2014) 5(11):2981-2988.
36. Gupta A, Bansal K, Marwaha M. Effect of high-molecular-weight component of Cranberry on plaque and salivary Streptococcus mutans counts in children: an in vivo study. J Indian Soc Pedod Prev Dent. (2015) 33(2):128-133.
37. Pak CY, Stewart A, Haynes SD. Effect of added citrate or malate on calcium absorption from calcium-fortified orange juice. J Am Coll Nutr. (1994) 13(6):575-577.
38. Hooper S, West NX, Sharif N, Smith S, North M, De'Ath J et al. A comparison of enamel erosion by a new sports drink compared to two proprietary products: a controlled, crossover study in situ. J Dent. (2004) 32(7):541-545.
39. Ismail AI, Sohn W, Lim S, Willem JM. Predictors of dental caries progression in primary teeth. J Dent Res. (2009) 88(3):270-275.
40. Gedalia I, Galon H, Rennert A, Biderco I, Mohr I. Effect of fluoridated citrus beverage on dental caries and on fluoride concentration in the surface enamel of children's teeth. Caries Res. (1981) 15(1):103-108.
41. Arnadottir IB, Saemundsson SR, Holbrook WP. Dental erosion in Icelandic teenagers in relation to dietary and lifestyle factors. Acta Odontol Scand. (2003) 61(1):25-28.
42. Hohensinn B, Haselgrubler R, Muller U, Stadlbauer V, Lanzerstorfer P, Lirk G et al. Sustaining elevated levels of nitrite in the oral cavity through consumption of nitrate-rich beetroot juice in young healthy adults reduces salivary pH. Nitric Oxide. (2016) 60:10-15.
43. Macpherson LM, Dawes C. Distribution of sucrose around the mouth and its clearance after a sucrose mouthrinse or consumption of three different foods. Caries Res. (1994) 28(3):150-155.
44. Parkar SG, Eady S, Cabecinha M, Skinner MA. Consumption of apple-boysenberry beverage decreases salivary Actinomyces naeslundii and their adhesion in a multi-species biofilm model. Benef Microbes. (2017) 8(2):299-307.
45. Zare Javid A, Maghsoumi-Norouzabad L, Ashrafzadeh E, Yousefimanesh HA, Zakerkish M, Ahmadi Angali K et al. Impact of Cranberry Juice Enriched with Omega-3 Fatty Acids Adjunct with Nonsurgical Periodontal Treatment on Metabolic Control and Periodontal Status in Type 2 Patients with Diabetes with Periodontal Disease. J Am Coll Nutr. (2018) 37(1):71-79.
46. Toumba KJ, Duggal MS. Effect on plaque pH of fruit drinks with reduced carbohydrate content. Br Dent J. (1999) 186(12):626-629.
47. Tahmassebi JF, Duggal MS. The effect of different methods of drinking on the pH of dental plaque in vivo. Int J Paediatr Dent. (1997) 7(4):249-254.
48. Piangprach T, Hengtrakool C, Kukiattrakoon B, Kedjarune-Leggat U. The effect of salivary factors on dental erosion in various age groups and tooth surfaces. J Am Dent Assoc. (2009) 140(9):1137-1143.
49. Murugesh J, Annigeri RG, Raheel SA, Azzeghaiby S, Alshehri M, Kujan O. Effect of yogurt and pH equivalent lemon juice on salivary flow rate in healthy volunteers - An experimental crossover study. Interv Med Appl Sci. (2015) 7(4):147-151.
50. Chapple IL, Milward MR, Ling-Mountford N, Weston P, Carter K, Askey K et al. Adjunctive daily supplementation with encapsulated fruit, vegetable and berry juice powder concentrates and clinical periodontal outcomes: a double-blind RCT. J Clin Periodontol. (2012) 39(1):62-72.
51. Levine RS, Nugent ZJ, Rudolf MC, Sahota P. Dietary patterns, toothbrushing habits and caries experience of schoolchildren in West Yorkshire, England. Community Dent Health. (2007) 24(2):82-87.
52. Watanabe M, Wang DH, Ijichi A, Shirai C, Zou Y, Kubo M et al. The influence of lifestyle on the incidence of dental caries among 3-year-old Japanese children. Int J Environ Res Public Health. (2014) 11(12):12611-12622.
53. Hung HC, Colditz G, Joshipura KJ. The association between tooth loss and the self-reported intake of selected CVD-related nutrients and foods among US women. Community Dent Oral Epidemiol. (2005) 33(3):167-173.
54. Bernabe E, Vehkalahti MM, Sheiham A, Aromaa A, Suominen AL. Sugar-sweetened beverages and dental caries in adults: a 4-year prospective study. J Dent. (2014) 42(8):952-958.
55. El Aidi H, Bronkhorst EM, Huysmans MC, Truin GJ. Multifactorial analysis of factors associated with the incidence and progression of erosive tooth wear. Caries Res. (2011) 45(3):303-312.
56. Aidi HE, Bronkhorst EM, Huysmans MC, Truin GJ. Factors associated with the incidence of erosive wear in upper incisors and lower first molars: a multifactorial approach. J Dent. (2011) 39(8):558-563.
57. West NX, Hughes JA, Parker DM, Moohan M, Addy M. Development of low erosive carbonated fruit drinks 2. Evaluation of an experimental carbonated blackcurrant drink compared to a conventional carbonated drink. J Dent. (2003) 31(5):361-365.
58. West NX, Hughes JA, Parker D, Weaver LJ, Moohan M, De'Ath J et al. Modification of soft drinks with xanthan gum to minimise erosion: a study in situ. Br Dent J. (2004) 196(8):478-481; discussion 467.
59. Hughes JA, West NX, Parker DM, Newcombe RG, Addy M. Development and evaluation of a low erosive blackcurrant juice drink. 3. Final drink and concentrate, formulae comparisons in situ and overview of the concept. J Dent. (1999) 27(5):345-350.
60. Horswill CA, Stofan JR, Horn MK, Eddy DE, Murray R. Effect of exercise and fluid consumption on salivary flow and pH. Int J Sports Med. (2006) 27(6):500-504.
61. Sauro S, Mannocci F, Piemontese M, Mongiorgi R. In situ enamel morphology evaluation after acidic soft drink consumption: protection factor of contemporary toothpaste. Int J Dent Hyg. (2008) 6(3):188-192.
62. Saeed S, Al-Tinawi M. Evaluation of acidity and total sugar content of children's popular beverages and their effect on plaque pH. J Indian Soc Pedod Prev Dent. (2010) 28(3):189-192.
63. Rugg-Gunn AJ, Maguire A, Gordon PH, McCabe JF, Stephenson G. Comparison of erosion of dental enamel by four drinks using an intra-oral applicance. Caries Res. (1998) 32(5):337-343.
64. Takayanagi A, Nomura T, Yamanaka S, Takaesu Y. A modified device for long term sampling of parotid saliva in various experimental conditions. Bull Tokyo Dent Coll. (1995) 36(2):69-73.
65. Warren JJ, Weber-Gasparoni K, Marshall TA, Drake DR, Dehkordi-Vakil F, Dawson DV et al. A longitudinal study of dental caries risk among very young low SES children. Community Dent Oral Epidemiol. (2009) 37(2):116-122.
66. Weber-Gasparoni K, Warren JJ, Reeve J, Drake DR, Kramer KW, Marshall TA et al. An effective psychoeducational intervention for early childhood caries prevention: part II. Pediatr Dent. (2013) 35(3):247-251.
67. Livny A, Sgan-Cohen HD. A review of a community program aimed at preventing early childhood caries among Jerusalem infants--a brief communication. J Public Health Dent. (2007) 67(2):78-82.
68. Ribeiro JS, Peralta SL, Salgado VE, Lund RG. In situ evaluation of color stability and hardness' decrease of resin-based composites. J Esthet Restor Dent. (2017) 29(5):356-361.
69. Briso AL, Fagundes TC, Gallinari MO, Moreira J, de Almeida L, Rahal V et al. An In Situ Study of the Influence of Staining Beverages on Color Alteration of Bleached Teeth. Oper Dent. (2016) 41(6):627-633.
70. Beck AL, Fernandez A, Rojina J, Cabana M. Randomized Controlled Trial of a Clinic-Based Intervention to Promote Healthy Beverage Consumption Among Latino Children. Clin Pediatr (Phila). (2017) 56(9):838-844.
71. Marshall TA. Preventing dental caries associated with sugar-sweetened beverages. J Am Dent Assoc. (2013) 144(10):1148-1152.
72. Bassiouny MA. Clinical observations of dental erosion associated with citrus diet and intake methods. Gen Dent. (2014) 62(1):49-55.
73. Bassiouny MA. Effect of sweetening agents in acidic beverages on associated erosion lesions. Gen Dent. (2012) 60(4):322-330; quiz 331-322.
74. Myklebust S, Espelid I, Svalestad S, Tveit AB. Dental health behavior, gastroesophageal disorders and dietary habits among Norwegian recruits in 1990 and 1999. Acta Odontol Scand. (2003) 61(2):100-104.
75. Kolker JL, Yuan Y, Burt BA, Sandretto AM, Sohn W, Lang SW et al. Dental caries and dietary patterns in low-income African American children. Pediatr Dent. (2007) 29(6):457-464.
76. Galon H, Gedalia I, Soudayee D, Pintz D. Fluoridated citrus beverage for the prevention of dental caries: 3 years of fluoride supply and 2 years cessation. J Int Assoc Dent Child. (1982) 13(2):73-77.
77. Mannerberg F. Effect of lemon juice on different types of tooth surface. A replica study in vivo. Acta Odontol Scand. (1962) 20:153-164.
78. Dimitrova M, Kukleva M. [Model for early childhood caries risks]. Stomatologiia (Mosk). (2008) 87(4):29-32.
79. Mathew S, Luke AM, Walia T, Masri AG, Jamal H, Pawar AM. Effect of Fruit Juices and Other Beverages on Loss of Tooth Structure. Braz Res Ped Dent Integ Clin. (2018) 18(1):e3888.
80. Vieira AR, Chung C, Raffensperger SK, Muluk P. Milk Reverts the Effects of an Enamel Erosive but Healthy Diet. Braz Res Ped Dent Integ Clin. (2018) 19(1):e3848.
